# Increased Low-Frequency Oscillation Amplitude of Sensorimotor Cortex Associated with the Severity of Structural Impairment in Cervical Myelopathy

**DOI:** 10.1371/journal.pone.0104442

**Published:** 2014-08-11

**Authors:** Fuqing Zhou, Honghan Gong, Xiaojia Liu, Lin Wu, Keith Dip-Kei Luk, Yong Hu

**Affiliations:** 1 Department of Radiology, the First Affiliated Hospital, NanChang University, Nanchang, Jiangxi, China; 2 Department of Orthopaedics and Traumatology, Li Ka Shing Faculty of Medicine, The University of Hong Kong, Pokfulam, Hong Kong; University of Toronto, Canada

## Abstract

Decreases in metabolites and increased motor-related, but decreased sensory-related activation of the sensorimotor cortex (SMC) have been observed in patients with cervical myelopathy (CM) using advanced MRI techniques. However, the nature of intrinsic neuronal activity in the SMC, and the relationship between cerebral function and structural damage of the spinal cord in patients with CM are not fully understood. The purpose of this study was to assess intrinsic neuronal activity by calculating the regional amplitude of low frequency fluctuations (ALFF) using resting-state functional MRI (rs-fMRI), and correlations with clinical and imaging indices. Nineteen patients and 19 age- and sex-matched healthy subjects underwent rs-fMRI scans. ALFF measurements were performed in the SMC, a key brain network likely to impaired or reorganized patients with CM. Compared with healthy subjects, increased amplitude of cortical low-frequency oscillations (LFO) was observed in the right precentral gyrus, right postcentral gyrus, and left supplementary motor area. Furthermore, increased z-ALFF values in the right precentral gyrus and right postcentral gyrus correlated with decreased fractional anisotropy values at the C2 level, which indicated increased intrinsic neuronal activity in the SMC corresponding to the structural impairment in the spinal cord of patients with CM. These findings suggest a complex and diverging relationship of cortical functional reorganization and distal spinal anatomical compression in patients with CM and, thus, add important information in understanding how spinal cord integrity may be a factor in the intrinsic covariance of spontaneous low-frequency fluctuations of BOLD signals involved in cortical plasticity.

## Introduction

It is unsurprising that the majority of cervical myelopathy (CM) studies focus on local changes in the spinal column or cord, because the main signs and symptoms of CM are primarily caused by damage to nerve fibers within the cervical spinal cord, particularly those in the lateral corticospinal tract [1]–[3]. However, cortical plasticity in response to CM may influence clinical symptoms, manifestations, and rehabilitation.

Advanced magnetic resonance imaging (MRI) is a useful tool for detecting CM-related cerebral alterations that may precede neuron metabolite abnormities [4], and hyper-activation in the primary motor cortex [5]–[7] or hypo-activation in the sensory cortex [6]. CM is a special spinal cord injury (SCI), which, secondary to degenerative diseases, is the most common spinal cord dysfunction disease. CM has similar local damage with SCI but different mechanisms of cortical alteration [8], [9]. In patients with CM, cerebral functional reorganization or plasticity secondary to neuronal damage in the spinal cord has been accepted as an important disease mechanism [4]–[6], [10]. However, the nature of intrinsic neuronal activity in the sensorimotor cortex (SMC), and the relationship between cerebral function and structural damage of the spinal cord in patients with CM are not fully understood. Thus, the characterization of changes in spontaneous neuronal functional activity of SMC and correlations with clinical and imaging indices may provide additional information about brain dysfunction in patients with CM.

In the current study, we chose the SMC (a hallmark region in CM) as a priori region of interest (ROI). The purpose was to assess alterations of regional cortical low-frequency oscillations (LFO) amplitude in the patients with CM using rs-fMRI. The amplitude of low frequency fluctuations (ALFF) was calculated to measure cortical LFO amplitude values of regional resting state functional MRI (rs-fMRI) time courses [11], [12]. Our hypothesis was that CM would alter the amplitude of oscillations of local neural activity in the SMC, which in turn would be related to the clinical status, or structural impairment of the spinal cord in patients with CM. Rs-fMRI data was acquired from 19 patients with CM and 19 age- and sex-matched matched healthy subjects. Cortical LFO amplitude (also named ALFF) was calculated, and was then compared across patients and controls and correlated with disease severity, duration, and spinal cord damage severity to assess clinical relevance.

## Results

### Clinical data profiling

Demographic and clinical data of the study groups is shown in Table 1. There were no significant differences between the groups with respect to age (*p* = 0.99) or sex (*p* = 0.95). The CM patient group presented loss of dexterity in the hands and gait dysfunction. There was a significant difference between the CM group and control group in Japanese Orthopaedic Association (JOA) scores and FA values in cervical cord.

**Table 1:** Demographic data and clinical measures scores for cervical myelopathy group and healthy controls.

Subject	Cervical myelopathy	Healthy controls	P-value
n	19	19	n/a
Age	49.63±7.36	49.46±7.21	0.99
Gender (male/female)	11/8	10/9	0.95
Handedness (right/left)	19/0	19/0	n/a
Duration of symptoms (month)	8.68±9.36	n/a	n/a
JOA scores	11.84±2.67	17±0	<0.0001
Motor upper	2.15±0.76	4±0	<0.0001
Motor lower	3.37±1.11	4±0	<0.0001
Sensory deficit	3.36±0.83	6±0	<0.0001
Bladder dysfunction	2.95±0.22	3±0	<0.0001
FA values			
FA values at the C2 level	0.601±0.046	0.665±0.047	0.0146
FA values at the severest level	0.507±0.071	0.657±0.026*	0.0007

n/a =  not applicable; JOA =  Japanese Orthopaedic Association; NDI =  Neck Disability Index; FA =  Fractional Anisotropy; C =  Cervical vertebra;

*mean FA values of whole cervical cord.

### ALFF/LFO amplitude alterations in the SMC in the CM group

**Figure 1:**
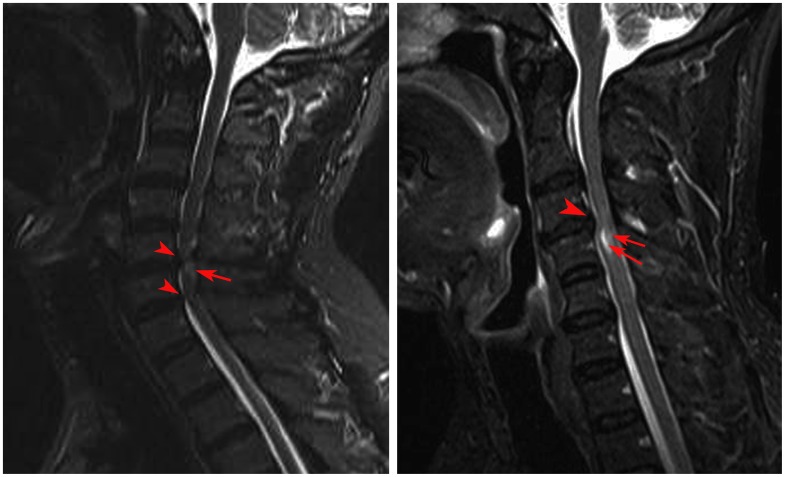
Two sample t-test analysis, and function of brain-structure in spinal cord relationship analysis. (a) ALFF/LFO amplitude differences between the CM and healthy subjects groups (CSM > Controls, p<0.05, AlphaSim corrected; cluster size ≥20). Warm colors indicate ALFF/LFO amplitude increases in patients with CM. T-score bars are shown on the right. (b) The correlation analysis results between the z-ALFF values of the right PreG, right PostG and the FA values at the C2 level of the CM patients. (C =  Cervical vertebra; CM =  Cervical myelopathy; P =  Posterior; PreG =  Precentral gyrus; PostG =  Postcentral gyrus; SMA =  Supplementary Motor Area; R =  Right hemisphere).

Figure 1a shows group-level CM vs. control group ALFF differences within the SMC. Compared with the control group, the CM group had a significantly higher ALFF (red-yellow spots in Figure 1a) in the right precentral gyrus (PreG), right postcentral gyrus (PostG), and left supplementary motor area (SMA). The t-value and the cluster size of the CM vs. control group ALFF differences are listed in Table 2.

**Table 2:** Significant ALFF/LFO amplitude difference between CM patients and healthy subjects.

Functional area	BA	Brain regions	Peak intensity-value	Number of voxels	Peak location (MNI)		
					x	y	z
CM patients > Health subjects							
Premotor cortex	BA6	Right Precentral Gyrus	3.396	41	57	0	15
		Left Supplementary Motor Area	3.595	80	−15	−6	63
Sensory cortex	BA2	Right Postcentral Gyrus	3.486	29	24	−42	57

(p<0.05, corrected for multiple comparisons, cluster size ≥20 voxels).

Notes: BA =  Brodmann area; ALFF =  Amplitude of low frequency fluctuations; MNI =  Montreal neurological institute; LFO =  low-frequency oscillations.

### Clinical associations with ALFF/LFO amplitude of the SMC in the CM group

Figure 1b and Table 3 show correlation results of z-ALFF values with CM group clinical measures. z-ALFF values in the right PreG (*R^2^* = 0.210, *p* = 0.048; *β* = −0.458, 95% confidence interval [CI]: −0.755 to −0.005) and right PostG (*R^2^* = 0.229, *p* = 0.038; *β* = −0.478; 95% CI: −0.766 to −0.030) showed a significant negative correlation with fractional anisotropy (FA) values at the C2 level. In contrast, there was no significant relationship between cortical z-ALFF values within the SMC and FA values at the most severe level (*p*: 0.141 to 0.7256), JOA score (*p*: 0.093 to 0.726), or disease duration (*p*: 0.394 to 0.842).

**Table 3:** Relationship between clinical status indices and the z-ALFF values in CM patients.

	Beta values (*P* value)		
	Right precentral gyrus	Right postcentral gyrus	Left supplementary motor area
Disease duration(month)	0.132(0.590)	0.207(0.394)	−0.049(0.842)
FA values in C2 level	−0.458(0.048)*	−0.478(0.038)*	−0.260(0.282)
FA values in the most severe level	−0.351(0.141)	−0.260(0.282)	0.086(0.7256)
JOA score	−0.086(0.726)	−0.112(0.647)	−0.397(0.093)

Notes: JOA =  Japanese Orthopaedic Association; FA =  Fractional Anisotropy; C =  Cervical vertebra.

*P<0.05, significant correlation between indices and the z-ALFF value.

## Discussion

In the current study, we showed increased cortical ALFF/LFO amplitude in the right PreG, the right PostG, and the left SMA in patients with CM. Furthermore, increased z-ALFF values in the right PreG and right PostG correlated with decreased FA values at the C2 level, which indicated increased oscillation amplitude in the SMC corresponding with the structural impairment in CM [3]. To our knowledge, this is the first study to investigate cortical LFO amplitude in a CM population. The findings show that higher cortical LFO amplitudes (meaning reorganization or plasticity) of the SMC seen on rs-fMRI are consistent with increased functional activation reported in previous studies [5]–[7].

Previous investigations have used task related fMRI to determine the cortical representation for upper and lower extremity function in CSM patients [5]–[7]. However, it is difficult to perform task fMRI test in most patients with CSM due to their motor dysfunction in hand motion or incompliance. In this study, ALFF/LFO amplitude is one of resting fMRI analysis techniques with task-free. The variances of ALFF/LFO amplitude could provide the intrinsic information base on bias input selection, temporally link neurons into assemblies, and facilitate synaptic plasticity [12], [13], while task related fMRI mainly reflect the response capability of neuron. As an important aspect of neural activity, some studies have confirmed that the ALFF method directly measures the LFO amplitude with high reliability and reproducibility both in the inter-session and intra-session fMRI scans [12], [14], [15].

On the altered spatial pattern, the results of increased ALFF/LFO amplitude in the motor cortex (right PreG and left SMA) are consistent with other functional neuroimaging studies [5]–[7], [16], and similar to motor cortical studies in SCI [9], [17]–[20]. Normally, the motor center is involved in the planning, control, and execution of voluntary movements through the spinal cord to muscles. In patients with CM, motor cortical activation (task fMRI) is larger compared with healthy subjects [5], [6], and is also larger in patients with SCI [18], [21]–[23]. ALFF/LFO amplitude reveals the local cortical intrinsic dynamic activity, which is associated with its connectivity [13] and is permissive to predict the specific task-evoked brain responses and behavioral performance [24]. Taken together, one explanation for the higher ALFF/LFO amplitude of the motor cortex in this study is that cortical reorganization or plasticity was initiated in response to decreased motor nerve conduction in the spinal cord. Horizontal intracortical axons and dendrites interconnect different movement representations of the motor cortex and likely serve an important role in neuroplasticity. The FA values of spinal cord was found as a promising and useful metric for assessing disease severity in CM [25]. Especially, the relative high FA at the C2 vertebra enables prediction of good surgical outcome [3]. In this study, the correlation between increased z-ALFF values of the right PreG and decreased FA values at the C2 level, it provide further evidence for cortical reorganization or plasticity as compensatory mechanisms distal to the structural impairment in the spine of patients with CM. Although the cortical ALFF/LFO amplitude and FA values coupling were only disclosed at C2 level, rather than the most severe level of compression. Superimposed on this background, it is reasonable to believe that increased CM-related LFO amplitude in motor cortex probably could corresponding to the structural impairment in the spinal cord, which may predict the severity of myelopathy and an indication of surgery. This may explain one mechanism in part of the patients with CM, who have distinct evidence of conspicuous cervical compression and degenerative demyelination, are able to perform motor activities with little or mild neurological deficits.

Increased LFO amplitude was also observed in the right PostG. The PostG is the location of the somatosensory cortex, the main sensory receptive area for the senses, and a projection to Brodmann's area 2 communicates size and shape. One of main symptoms of CM is limb numbness and neck pain, in which increased abnormal sensory input drive is followed by higher oscillation amplitude of neuronal activity. Thus, another interpretation of increased cortical LFO amplitude in the PostG could be increased modulation of cortical activity occurring in patients with CM. This could potentially be associated with ongoing plasticity and cortical remapping, which are widely reported in patients with SCIs [18], [26] and animal models [22]. It should be noted that lower (task-related) activation in the PostG gyrus in patients with CM compared with healthy subjects has been previously reported [6]. The discrepancy between studies might be explained by the fact that the physiological mechanisms of task versus resting state fMRI are different. The relationship between z-ALFF values of the right PostG and decreased FA values at the C2 level is consistent with the concept that increased cortical LFO amplitude is correlated with the structural impairment in CM.

Another alternative interpretation of increased cortical LFO amplitude of the SMC could therefore be a dis-inhibitory influence at a local regional level, interrupted by a loss of afferent or efferent fibers, which could facilitate cortical reorganization through the disinhibited connections. Indeed, animal studies have shown that γ-aminobutyric acid maintains inhibitory interconnections [27], [28]. It should be noted that lower metabolite levels in the motor cortex of patients with CM [4] and SCIs [21] compared with controls has also been previously reported. This may be explained by local inhibitory neuronal damage due to higher levels of neuronal metabolic activity at baseline.

Interestingly, increased cortical LFO was observed in the SMA of the CM group, although it was not a symmetrical distribution. The inconsistent maturity of inter-hemispheric is a possible explanation, reflected in the availability of cortical neuron pools and the ability of existing corticospinal tracts to activate spinal motor pools to activate spinal pools in patients with CM [5].

However, no significant correlations were observed between cortical z-ALFF values within the SMC and JOA scores and disease duration. Although the JOA system is recommended but there are some clinical disadvantages, such as sensitiveness, effectiveness, and ignorance of its physical functions of the cervical spine (e.g. range of motion of the neck, pain) [29]. In brain studies of CM patients, no direct correlation was observed between JOA score and motor-related activation [6], neuronal metabolite ratios [4]. The lack of a correlation in our study also suggests that the various changes of clinical function may be mainly dominated by the local insult to the cord, and not regional ALFF/LFO amplitude alterations in the cortex.

One limitation of the present study is that our SMC mask was selected from previous literature [30]. While other SMC masks may identify different ALFF/LFO amplitude levels in undiscovered regions, using different mask should not change the overall results reported in this paper. In future studies, a whole brain analysis and a post-operative decompression study would be useful to add to findings reported here. Another, as an explorative study of correlation analysis between the cortical z-ALFF values and clinical measures in patients with CM, we did not use multiple comparisons correction. The sensitivity difference of regions in clusters of the SMC for predicting different clinical measures may also be caused by the moderate sample size, as Table 3 shows that some regions showed a trend with a moderate *r* value for correlations with clinical measures. Regarding these facts of the physiology basis of ALFF/LFO amplitude and its association with structural damage at the C2 level, future work will determine whether alterations in cortical ALFF/LFO amplitude could predict or limit functional recovery following spinal decompression surgery.

Moreover, the present study did not investigate the laterality of the ALFF/LFO amplitude and the CSM related neurological symptoms. Considering the complexity of symptoms, multilevel and multiformity of compression region [8], it needs further study with large scale clinical trial and advanced statistical analysis to investigate this issue.

## Conclusions

In summary, this study showed that increased cortical LFO amplitude correlated with the spinal cord structural impairment at C2 level in patients with CM. Our findings provide further evidence of sensorimotor cortical ALFF/LFO amplitude abnormalities in patients with CM. These findings suggest a complex and diverging relation of cortical functional reorganization and distal spinal anatomic compression in patients with CM and, thus, aid understanding about how to link neuronal alteration and damage of the spinal cord.

## Materials and Methods

### Participants

This study was approved by the institutional review board of the First Affiliated Hospital, Nanchang University, China. A written informed consent was obtained.

**Figure 2:**
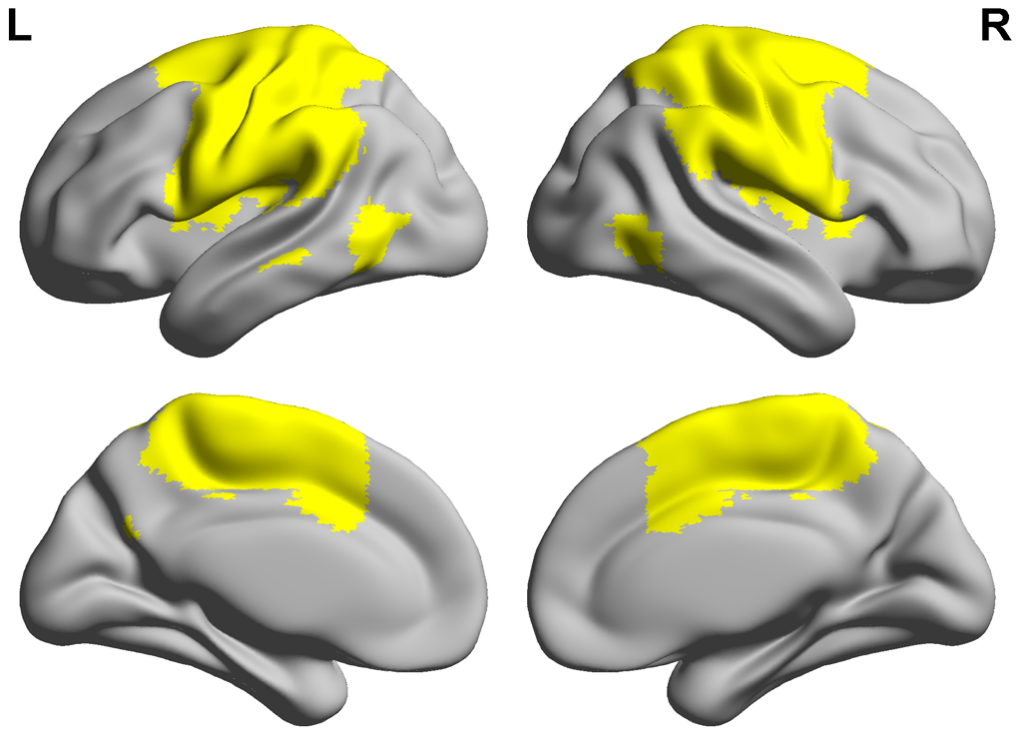
The representative images showing sagittal T2W images in the myelopathic cord. The red arrowhead and arrow indicates the cervical compression and degenerative demyelination, respectively.

Nineteen right-handed patients with degenerative CM (8 females and 11 males; mean age 49.63±7.36 years (mean ± standard deviation)) were recruited at the First Affiliated Hospital of NanChang University from May 2013 to November 2013. The mean duration of symptoms from disease onset to the date of MRI examination was 8.68±9.36 months. The clinical severity of myelopathy was evaluated using the Japanese Orthopaedic Association (JOA) score system [29] (11.84±2.67). Inclusion criteria of patients included: (1) volunteer to enroll in the study; (2) clear evidence of cord compression on a cervical spine MRI (Fig. 2), such as cervical spondylosis, or an ossified posterior longitudinal ligament, and (3) demyelination with hyper-intensity of cord on T_2_WI. Two radiologists determined spinal cord compression when the cord surface was clearly indented or cord diameter was narrowed by compression. Exclusion criteria included: (1) refusal by the patient to enroll; (2) trauma or infection related to cord compression; and (3) other neurological disorders such as multiple sclerosis, or a history of trauma.

Nineteen right-handed age- and sex-matched healthy subjects with no previous clinical history of CM or neurological disease were recruited. Based on rs-fMRI data, participants with maximum displacements in one or more of the orthogonal directions (x, y, z) of >2 mm or a maximum rotation (x, y, z) >2.0° in max head motion were excluded.

### Image acquisition

MRI scans were performed using a 3.0 Tesla MRI scanner (Trio Tim, Siemens, Erlangen, Germany). Subjects were instructed to their keep eyes closed and not to think about anything in particular, and not to fall asleep. A total of 240 time point rs-fMRI brain images (duration  = 8 min) were acquired using a standard T_2_*-weighted gradient echo sequence with the following parameters: repetition time (TR)/echo time (TE)  = 2000/30 ms, field of view (FOV) = 200×200 mm, matrix  = 64×64, 30 interleaved axial slices with 4-mm thickness with an inter-slice gap of 1.2-mm. Sagittal and axial conventional T_1_W, T_2_W and T_2_-FLAIR images were acquired in the brain and cervical spinal cord for diagnosis in each subject. Additional DTI images using a spin-echo single-shot echo-planar sequence were acquired to evaluate cervical structural damage severity [3] (TR/TE = 5000/106 ms; number of excitations (NEX)  = 2; matrix  = 128×124; FOV = 128×124 mm; slices  = 16; slice thickness  = 5 mm; orientation  =  axial; 20 nonlinear diffusion weighting gradient directions with b = 600 s/mm^2^ and 1 additional image without diffusion weighting [i.e., b = 0 s/mm^2^]). The image slice planning was the same as the anatomical axial T_1_W and T_2_W images, covering the cervical spinal cord from C1 to C7.

### Rs-fMRI data preprocessing

The first 10 time points were discarded to allow the MR signal to reach steady state and participants to get used to the scanner noise. Rs-fMRI images were slice-timing corrected, motion corrected, and spatially realigned to adjust the time series of images using the Data Processing Assistant for Resting-State fMRI Advanced Edition (DPARSFA) V2.2 (http://www.restfmri.net) [31] running in Matlab 7.14.0 (Mathworks, Natick, MA, USA). The images were then registered with the high-resolution T1 image using SPM8 (http://www.fil.ion.ucl.ac.uk/spm/software/spm8/), normalization to Montreal Neurological Institute 152 (MNI152) space with 3×3×3 mm^3^ re-sampling. Spatial smoothing was performed using a 6-mm full-width-half-maximum Gaussian kernel, and temporal band-pass filtering (0.01<f<0.08 Hz) were performed to reduce the effects of low-frequency drift and physiological high-frequency noise [31].

### Cortical ALFF/LFO amplitude computing

**Figure 3:**
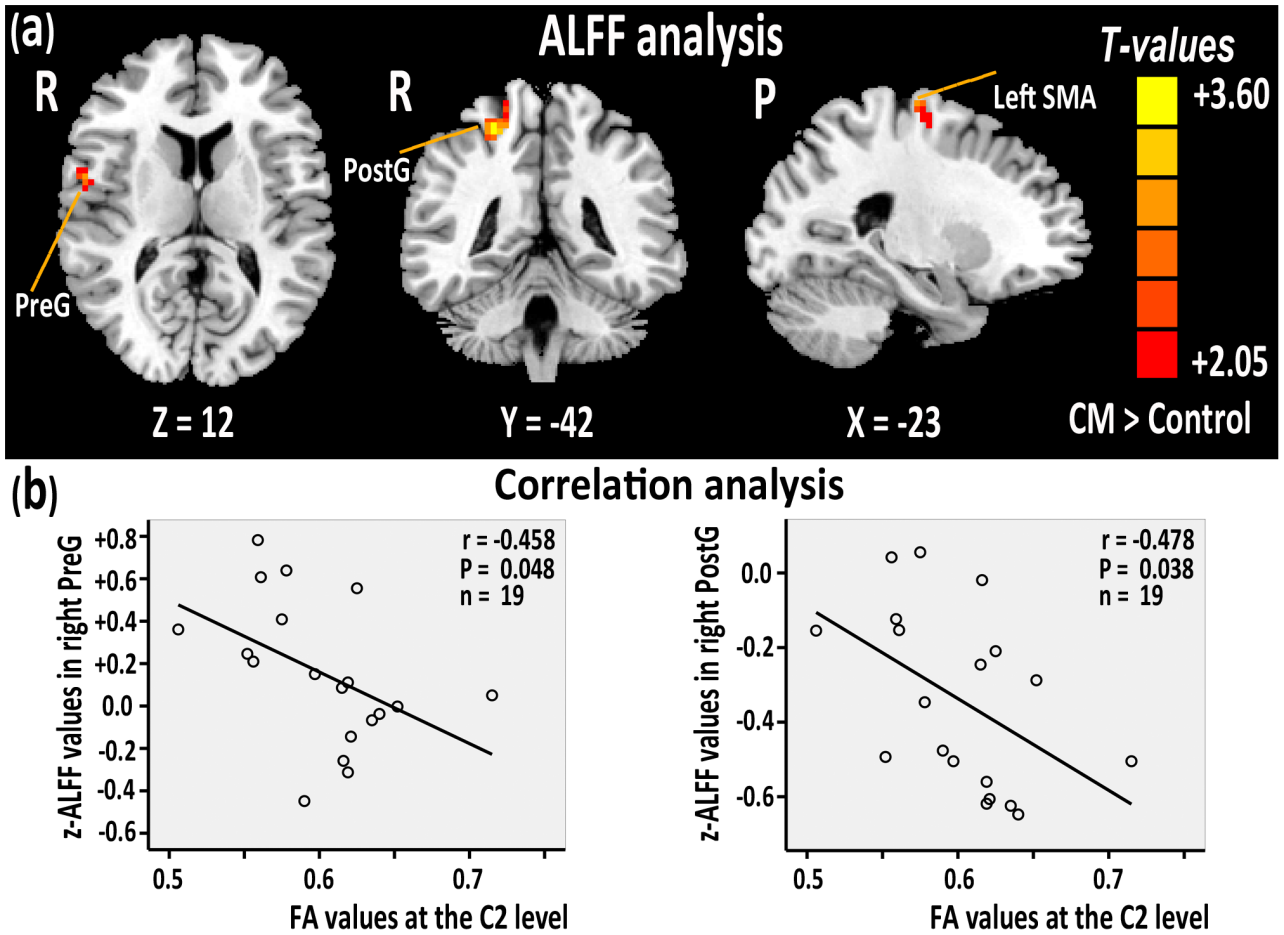
Illustration of the sensory-motor cortex (SMC) mask used in this study (L =  left hemisphere; R =  right hemisphere). Functional SMC mask generated with independent component analysis (ICA) was obtained from the Medical Image Analysis (MIA) Lab (Allen et al., 2011). The SMC mask consists of the bilateral primary motor cortex, the supplementary motor area (SMA), and the bilateral primary somatosensory cortex.

The ALFF values were calculated to measure cortical LFO amplitude values within the SMC [11]. The procedures used to obtain individual ALFF maps within a functional SMC mask [30], [32] (Fig. 3) were implemented using the DPARSFA toolkit, similar to that described previously [31]. The ALFF values were z-transformed with Fisher's z transformation and were used for subsequent group-level analysis, which was visualized using the REST Viewer (http://www.restfmri.net).

### Fractional anisotropy (FA) metrics calculation in the cervical spinal cord

FA metrics were calculated in DTI native space for each subject using the Diffusion Toolkit, which is one component of the TrackVis (http://www.trackvis.org/) software package. Regions of interest (ROIs) were typically placed at the C2 vertebra level and the level of most severe cervical canal stenosis.

### Statistics analysis

A two-sample t-test was performed to assess group level ALFF differences. The statistical significance of the CM vs. controls group difference was determined using a Monte Carlo simulation (AlphaSim; single voxel p = 0.05, FWHM  = 6 mm, 10,000 simulations, using the SMC mask [7070 voxels]) [33] combined with cluster size ≥20 voxels, this correction was conducted using the AlphaSim program embedded into the REST package (http://www.restfmri.net). Linear regression was performed to assess the association of ALFF/LFO amplitude to distinct clinical measures, including disease duration, JOA score, and mean FA values in the cervical cord (spinal cord damage severity [3]). SPSS v13.0 was used for statistical analyses (SPSS Inc., Chicago, IL, USA).
