# Correction: Increased Low-Frequency Oscillation Amplitude of Sensorimotor Cortex Associated with the Severity of Structural Impairment in Cervical Myelopathy

**DOI:** 10.1371/journal.pone.0112588

**Published:** 2014-10-30

**Authors:** The PLOS ONE Staff

The images for Figures 1, 2 and 3 are switched. The image that appears as Figure 3 should be Figure 1, the image that appears as Figure 1 should be Figure 2, and the image that appears as Figure 2 should be Figure 3. The figure legends appear in the correct order.

**Figure 1:**
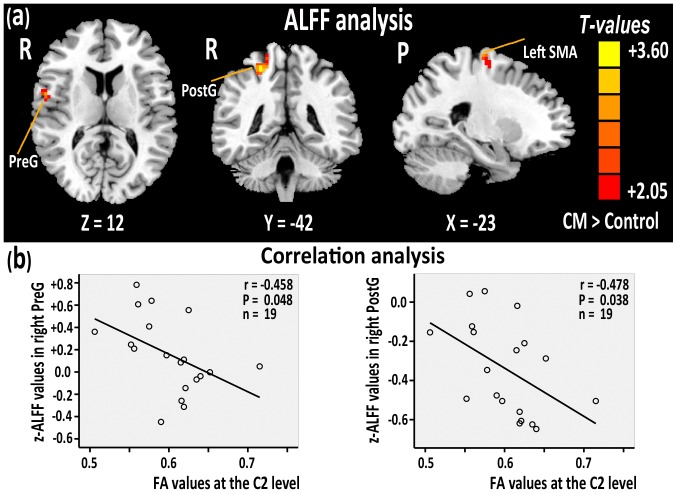
Two sample t-test analysis, and function of brain-structure in spinal cord relationship analysis. (a) ALFF/LFO amplitude differences between the CM and healthy subjects groups (CSM> Controls, p<0.05, AlphaSim corrected; cluster size ≥20). Warm colors indicate ALFF/LFO amplitude increases in patients with CM. T-score bars are shown on the right. (b) The correlation analysis results between the z-ALFF values of the right PreG, right PostG and the FA values at the C2 level of the CM patients. (C  =  Cervical vertebra; CM  =  Cervical myelopathy; P  =  Posterior; PreG  =  Precentral gyrus; PostG  =  Postcentral gyrus; SMA  =  Supplementary Motor Area; R  =  Right hemisphere).

**Figure 2:**
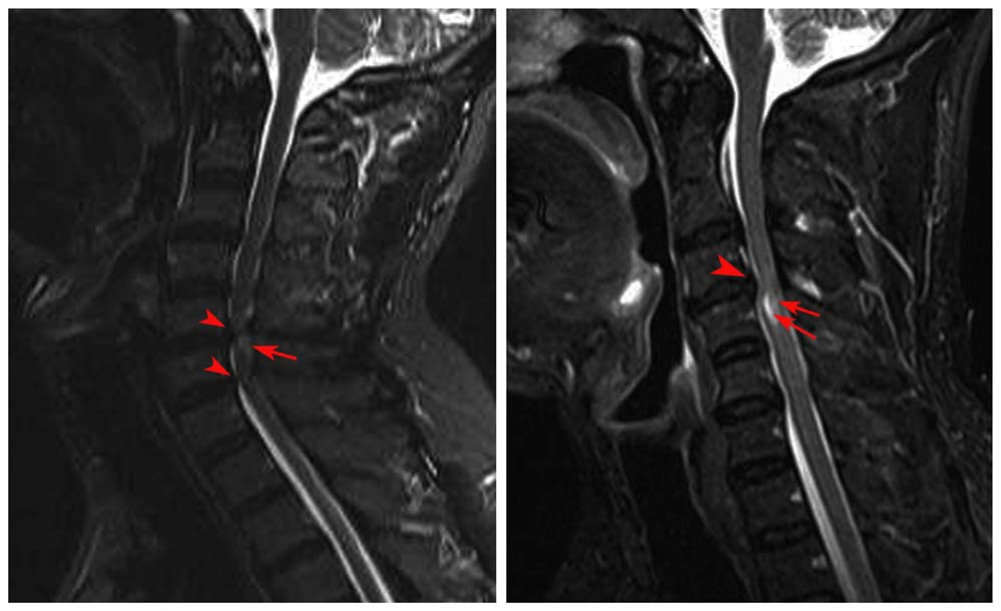
The representative images showing sagittal T2W images in the myelopathic cord. The red arrowhead and arrow indicates the cervical compression and degenerative demyelination, respectively.

**Figure 3:**
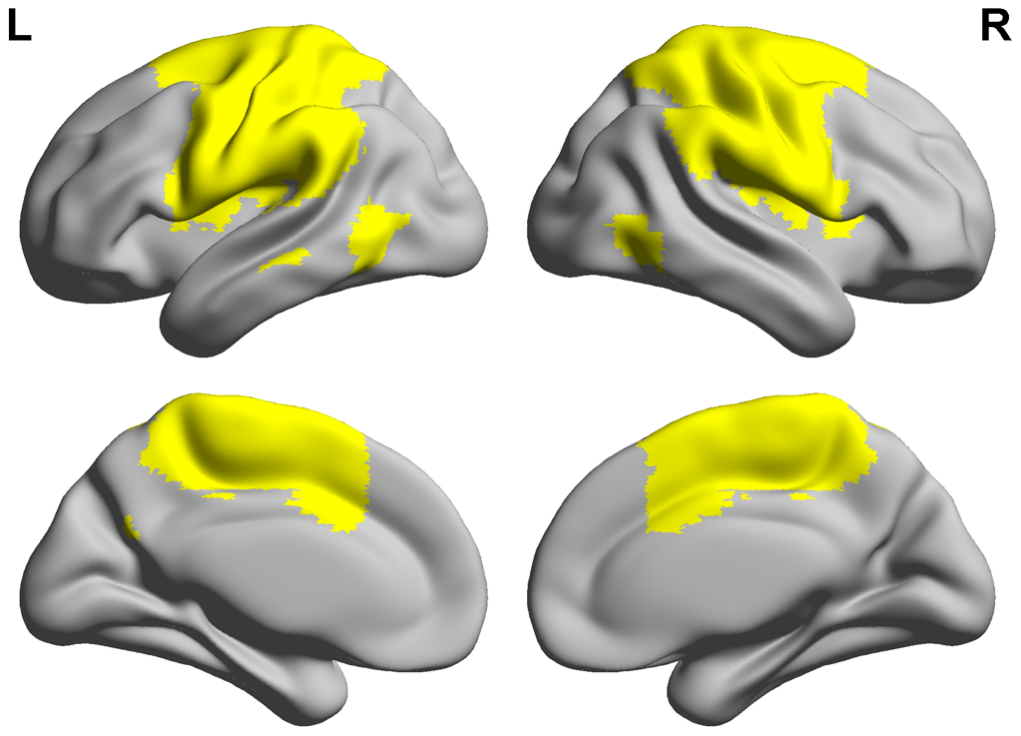
Illustration of the sensory-motor cortex (SMC) mask used in this study (L  =  left hemisphere; R  =  right hemisphere). Functional SMC mask generated with independent component analysis (ICA) was obtained from the Medical Image Analysis (MIA) Lab (Allen et al., 2011). The SMC mask consists of the bilateral primary motor cortex, the supplementary motor area (SMA), and the bilateral primary somatosensory cortex.
